# Bringing Structure to Cell Biology with Cryo-Electron Tomography

**DOI:** 10.1146/annurev-biophys-111622-091327

**Published:** 2023-05-09

**Authors:** Lindsey N. Young, Elizabeth Villa

**Affiliations:** 1 Department of Molecular Biology, University of California, San Diego, La Jolla, California, USA;; 2 Howard Hughes Medical Institute, University of California, San Diego, La Jolla, California, USA

**Keywords:** cryo-electron tomography, in situ structural biology, quantitative cell biology, biophysical modeling, molecular architecture

## Abstract

Recent advances in cryo-electron microscopy have marked only the beginning of the potential of this technique. To bring structure into cell biology, the modality of cryo-electron tomography has fast developed into a bona fide in situ structural biology technique where structures are determined in their native environment, the cell. Nearly every step of the cryo-focused ion beam-assisted electron tomography (cryo-FIB-ET) workflow has been improved upon in the past decade, since the first windows were carved into cells, unveiling macromolecular networks in near-native conditions. By bridging structural and cell biology, cryo-FIB-ET is advancing our understanding of structure–function relationships in their native environment and becoming a tool for discovering new biology.

## INTRODUCTION

Since the 1940s, direct visualization into the inner working of a cell by conventional transmission electron microscopy (TEM) has facilitated discoveries and generated both new hypotheses and awe as researchers peered directly into the complex and heterogeneous cellular environment for the first time (154). TEM experiments on cells that were fixed, permeabilized, and stained, a process referred to as conventional TEM, gave way to high-pressure freezing (HPF) methods and freeze-substitution, which allowed researchers to inch closer to native conditions and better visualize a cell’s interior. Separately, the development of direct electron detector devices (DDDs) and new image processing algorithms precipitated the resolution revolution in single-particle cryo-electron microscopy (cryo-EM) (81). These technical developments further facilitated high-resolution 3D reconstructions of in vitro samples determined, not by combining thousands of 2D projections of different particles of the same kind (single-particle cryo-EM), but from projections acquired at multiple orientations or tilts of the same sample (tomography) (101, 127). The next hurdle was to visualize these macromolecules directly in cells (in situ or in cellulo), without any fixation, sectioning, or other perturbations. How could one achieve this? By borrowing techniques from materials science and by operating under cryogenic temperatures, researchers carved out a whole new field in which they could open windows directly into the complex cellular environment using focused ion beam (FIB) milling (94). The preservation of high-resolution information created a new field of structural cell biology studies in which subtomogram analysis, combined with other methods such as integrative modeling, has expanded how structure–function relationships can be explored and investigated (8, 94).

## BACKGROUND

Investigating how individual macromolecules organize to perform all functions in the cell, e.g., cellular growth, division, and migration, requires understanding how individual small players act in concert, resulting in emergent properties that accomplish larger tasks. Macromolecular complexes are roughly tens of nanometers across (102), and their interactions are governed by chemical bonds, electrostatic interactions, hydrogen bonds, and van der Waals forces. These proteins and macromolecular complexes then assemble to form structures that are micrometers in size (e.g., organelles, filaments). Investigating structure–function relationships in situ requires the ability to image across multiple length scales. Investigating how these distinct structures function in their native environment requires the ability to visualize macromolecules that are minimally perturbed. By imaging across multiple length scales (from angstroms to microns), an electron microscopist can investigate assemblies at the molecular level and also define relevant spatial relationships—all within a single cell. Furthermore, new discoveries can be made via electron microscopy (EM) because the interaction between the electron beam and the sample occurs for all atoms in the sample that are subsequently captured in the resulting image. This is in contrast to fluorescence microscopy, in which prior knowledge and labeling of key macromolecules are required for further investigation of their molecular function, and only labeled molecules are captured in the image. Essentially, an electron beam sees all.

Cryo-electron tomography (cryo-ET) is a modality of TEM in which a flash-frozen sample is imaged at multiple orientations by tilting the sample. The resulting collection of images, or tilt series, is aligned to reconstruct the sample in three dimensions into a tomogram, where the intensities in a sample are roughly proportional to the mass of the underlying atoms (159). From the tomogram, one can extract specific regions containing macromolecules of interest. These subtomograms can then be extracted, aligned, averaged, and further analyzed to obtain 3D structures of molecular complexes, a technique called subtomogram analysis (STA). In a proof of the principle that high-resolution information can be recovered from in situ data, a recent cryo-ET reconstruction of a ribosome stalled during protein translation was resolved to 3.4 Å (137). However, as only transmitted electrons are detected, there is a strict thickness limitation to this methodology. The mean free path of 300 keV electrons was determined to be 280 nm for free vesicles in cryogenic conditions (66, 97, 119, 162). Larger thicknesses result in multiple interactions of the electrons with the sample, dramatically reducing the signal-to-noise ratio (SNR) in the images. As most cells are thicker than 500 nm, samples need to be thinned. In the past decade, this limitation was overcome by cryo-FIB milling (141, 144). This technique ablates away most of the cellular material, leaving behind a window of several to tens of microns in *x* and *y*, with a thickness of approximately 80–300 nm. Cryo-FIB-ET is the highest-resolution technique available to image macromolecules inside cells, and an ever increasing number of landmark discoveries have been made in the past decade using this technology (1, 3, 47, 48). In this review, we describe the cryo-FIB-ET workflow, the recent advances in the field (sample preparation, data acquisition, and analysis),the integration with other methods, considerations for designing a successful project, and an outlook for the field.

## THE CRYO-FOCUSED ION BEAM MILLING AND ELECTRON TOMOGRAPHY WORKFLOW

**Figure 1:**
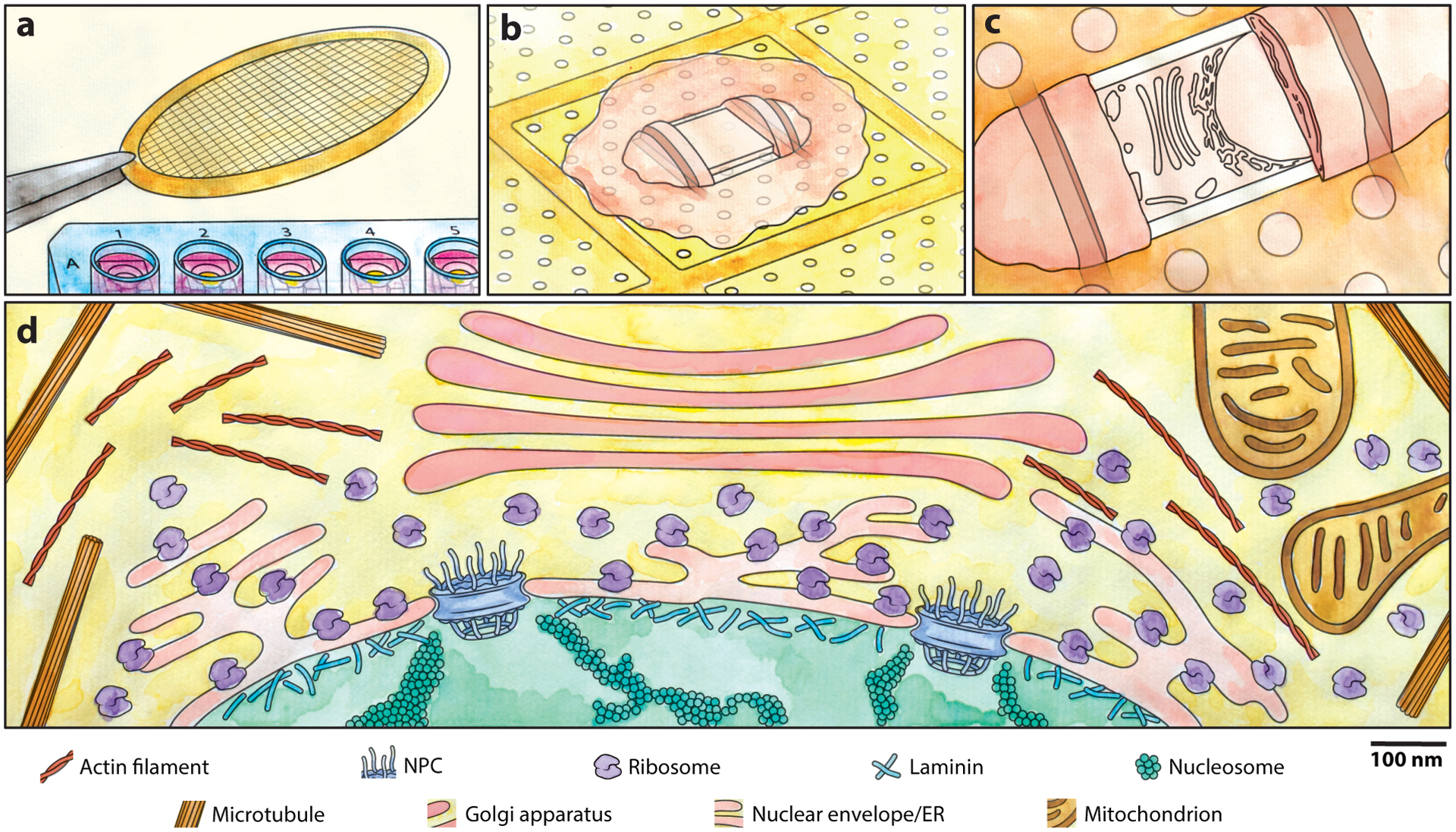
Cryo-FIB-ET workflow. (*a*) Adherent cells can be cultured on EM grids, allowing unique lab-on-a-grid experiments. (*b*) A thin window or lamella is carved into an adherent grid grown on an EM grid via an FIB under cryogenic conditions. Expansion joints (trenches) on either side of the lamella are made to improve mechanical stability by offering points to allow flexing. (*c*) The lamella is imaged by TEM under cryogenic conditions at multiple tilt angles to facilitate a 3D reconstruction of the molecules within the lamella. (*d*) The complex and heterogeneous interior of a cell can be imaged at the highest achievable spatial resolution, resolving individual macromolecules (ribosomes, the NPC, nucleosomes), filaments (microtubules, actin, lamin), and organelles (mitochondria, ER, nucleus, Golgi). Abbreviations: cryo-FIB-ET, cryo-focused ion beam milling and electron tomography; EM, electron microscopy; ER, endoplasmic reticulum; FIB, focused ion beam; NPC, nuclear pore complex; TEM, transmission electron microscopy. Figure copyright 2023 Dorotea Fracchiolla, adapted with permission.

Most commonly, cultured cells are grown or deposited onto an EM grid, and the sample is then rapidly frozen into a noncrystalline, glass-like vitrified state by plunging it into liquid ethane cooled to liquid nitrogen temperature. The result is preservation of macromolecular structures in their cellular networks in near-native conditions (40, 72). The sample is then transferred under cryogenic conditions (liquid nitrogen temperature) to a dual beam microscope equipped with an FIB and a scanning electron microscope (SEM); the FIB-SEM is routinely used within materials science for microstructural analysis, chip editing, nanomachining, and sample preparation for TEM (61). For vitrified cells, regions above and below the region of interest are selectively ablated as the FIB rasters across, removing cellular material via the interaction between an ion source, typically of gallium, and the sample in a process referred to as milling (94, 145). The resulting lamella is milled to a thickness of 80–300 nm (94, 145). The sample is transferred to a TEM, and regions of interest within the lamellae are imaged at different tilt angles to collect a tilt series that is later aligned and reconstructed computationally to generate a 3D volume or tomogram. The resulting tomogram shows the interior of the cell in three dimensions at the highest achievable resolution without fixation or perturbation (see Figure 1). Detailed workflows for cryo-FIB milling and electron tomography (cryo-FIB-ET) are available (82, 123, 143).

## ADVANCES IN THE CRYO-FOCUSED ION BEAM WORKFLOW

Recent improvements in the cryo-FIB-ET workflow have dramatically increased sample throughput, experimental handling, and improved data collection. For adherent cells, the first step in this workflow is seeding cells on an EM grid. Adaptation of micropatterning to EM grids enabled the control of the position, size, and shape of adherent cells on the EM grid (138). Added to this, 3D-printed holders for EM grids can minimize grid handling mishaps (49). It is unfortunate, after preparing a lamella, to discover that it is not suitable for high-resolution TEM data acquisition. This unsuitability could be due to lamellae being too thick, broken, badly vitrified, or contaminated during transfers, among others. Recent advances in the FIB milling process have resulted in more optimal lamellae that are more stable, have reduced ice contamination, and notably can be generated through automation. The stability of the lamella has been made more robust through stress-reducing expansion joints, or trenches, around the lamella site (157). A cryo-glove box was developed to reduce contamination during sample transfer (136). Incubating samples in 10% glycerol improved the vitrification of large samples, i.e., the peripheral nervous system tissues from *Drosophila* (7). Another challenge is that milling can result in curtains due to differential sputtering rates in cellular compositions, ice contamination, or milling at high currents for an extensive period of time (82, 123). Platinum deposition and sputtering alleviate some curtaining effects, as described below. To alleviate persistent curtaining and improve visual interpretability, curtain artifacts can be removed in silico, with regions of interest targeted on the postprocessed image (7). Finally, through FIB milling automation, sample throughput has improved, and a user is now able to generate up to 20 lamellae in a session through software automation (79, 80, 136, 163).

Newer methods for dealing with a diverse set of samples have expanded what can be imaged by this technique. Tissues, or other thick specimens, cannot be fully vitrified by plunge freezing due to inadequate heat transfer (133) but can be vitrified through HPF. After HPF, a section of the specimen is carved using the FIB, lifted out from the sample, transferred to a new grid placed in the same shuttle within the FIB-SEM instrument, and then further thinned down to generate a 100–300 nm lamella through a method referred to as lift out (93). A recent study used in situ sputtering from the micromanipulator to enable cryogenic preparation of specimens for atom probe tomography by FIB, enabling the analysis of silicon content within a sample (39).

As an alternative to lift out, the inventive waffle method makes cryo-FIB-ET more amenable to thick specimens; specimens that orient preferably on an EM grid, e.g., bacteria that lie along their long axis; and specimens that require a high local concentration, e.g., cells that need to grow to confluency (75). In this method, the sample is applied to the backside of an EM grid and is then high-pressure frozen, thereby achieving high uniform density, which facilitates cells orienting in multiple directions (75).

Recently, the types of ion sources used during the cryo-FIB milling process have been expanded to include plasma from gases including oxygen, nitrogen, argon, and xenon. Initial studies suggest that these gases may induce less damage from the ion beam and lead to higher-resolution data (12, 129).

### Data Collection

**Figure 2:**
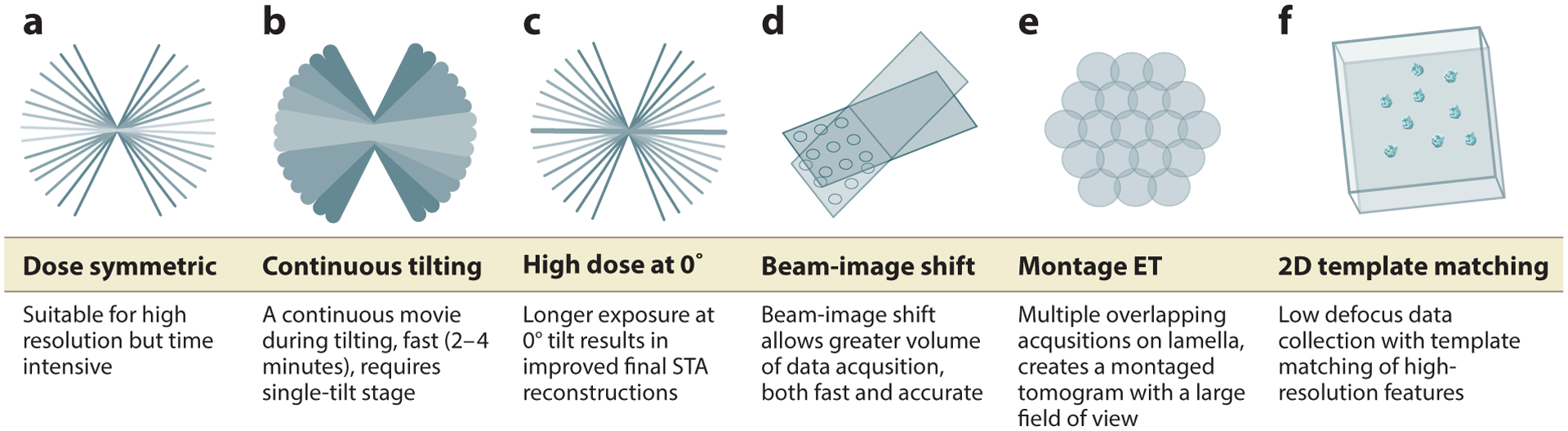
Data collection methods. What data collection method is best for your data? It depends. (*a*) In the dose-symmetric tilt series, data collection alternates between positive and negative tilts. (*b*) In continuous fast tilting, data are continuously acquired. (*c*) In hybrid STA-SPA, a high-dose image is collected at 0°, followed by implementation of the dose-symmetric method. (*d*) In beam-image shift, the beam is shifted from a central focus and tracking to targeted areas of acquisition, facilitating the collection of multiple tilt series simultaneously. (*e*) In a montage collection series, the entire lamella is imaged to create one large tomogram. (*f*) In 2D template matching, one single projection image is acquired at 0°, and high-resolution features of a target are matched to the data. Abbreviations: ET, electron tomography; SPA, single-particle analysis; STA, subtomogram analysis.

After producing a beautiful (or at least usable) lamella, the user must decide on a data collection scheme (Figure 2). A tilt series is a collection of images of a sample at different tilt angles. Each of these images is called a projection, as it collapses the 3D information of the sample along the optical path of the microscope into two dimensions (58). Because biological macromolecules are low atomic weight, they are considered to be weak-phase objects, as the generated contrast is weak. Image contrast is achieved by shifting the focal plane via defocusing the objective lens (62). This image defocus, along with other factors, introduces variations in the image described by the contrast transfer function (CTF) that can be corrected to retrieve a more accurate representation of the sample (51). Tilt series acquisition that produces a high-accuracy reconstruction in three dimensions relies on several factors, including stage and sample stability; lamella thickness; location of the feature of interest within the lamella; and, once the data are acquired, the ability to align each projection in the tilt series and CTF correct it. During data collection, additional images are typically acquired to ensure that the field of view remains centered in the area of interest (*XY* tracking) and at the same defocus (*Z*). Subsequently, to align the tilt series accurately for 3D reconstruction, features in the images of the tilt series must be followed from one image to the next (fiducials). Gold beads are electron dense and are utilized extensively for in vitro cryo-ET; these fiducials enable accurate focus and tracking during collection and tilt series alignment post-collection (147). In most cases, membranes and other features present in the cells can be used as fiducials for tilt series alignment. In one attempt to improve alignments for in situ studies, researchers added gold beads to the cell culture media; these gold beads were endocytosed, becoming fiducials within the lamella (13).

How the tilt series should be collected depends on several factors, including the scientific question, how the data will be analyzed, and sample quality. Software packages offer flexibility in tilt series acquisition; these include the academic packages SerialEM and Leginon (98, 134) and the commercial packages Tomography 5 (Thermo Fisher Scientific), Latitude (Gatan), and Recorder (JEOL). These packages allow the user to control parameters such as acquisition scheme, tilt range, tilt increment, dose, magnification, and target defocus. SerialEM is a broadly used and reliable package that is ideal to test new schemes while offering out-of-the box solutions, including automated tomography data collection (98). National Institutes of Health funding and user-contributed support of SerialEM facilitates such innovation by providing a platform on which users can develop and disseminate their own schemes for data collection.

A major consideration when acquiring data is the accumulated radiation damage to target areas of acquisition (65). When collecting 2D data [as in single-particle analysis (SPA) and 2D template matching, introduced below)], a single movie is acquired with a total fluence (commonly referred to as dose) of 60–120 e^−^/A^2^. In tomography, the total dose that a sample experiences has to be distributed among many images in the tilt series. The choice of how to distribute the dose determines how well the individual images can be aligned to others in the tilt series and which images have the most information: The projections generated from early stages in data collection will contain the least amount of radiation damage and the best-quality high-resolution information. A variety of tilt series acquisition schemes are available depending on such considerations.

Collection schemes include continuous collection, in which the sample is tilted to the maximum tilt angle on one side (e.g., –70°) and then toward the maximum tilt angle on the other side (e.g., +70°). The benefits of this scheme are speed of data acquisition and minimal stage perturbations. However, when acquiring a tilt series, the effective thickness of the sample increases as the sample tilts from 0° to 70° because there is more material for the beam to pass through at high tilts (the thickness increases as 1/cos of the tilt angle). Thus, the detriment of continuous collection is that the initial dose, when the sample is most undisturbed by radiation, is spent on high-tilt images, where the sample will be the thickest, resulting in a lower-contrast image. A commonly used bidirectional collection scheme starts at 0° (or at the angle where the lamella is thinnest, compensating for its pretilt), moves toward the highest angle in one direction, and then returns to the initial position, collecting until it reaches the maximum tilt angle in the other direction. A bidirectional tilt series will result in two halves, and since the adjacent projection images at the lower tilts will have experienced different amounts of accumulated dose, there is often a problem aligning the tilt series. Additionally, since the sample is thinnest at the low tilt angles, it is at these angles that the images can be attained at high resolution; however, in the bidirectional scheme, at low tilt angles on one side, the sample has received significant electron dose, and thus high-resolution information is lost (139). To maximize initial dose at lower tilts while still producing a smooth reconstruction, Hagen et al. (67) developed the dose-symmetric (DS) scheme, also known as the Hagen scheme, in which acquisition begins at 0° (or the lamella pretilt) and alternates between positive or negative tilt angles along the tilt range. A thorough comparison among these schemes via STA of in vitro data concluded that the DS scheme resulted in the highest attainable resolution (139).

Beyond optimizing for individual tilt series, new collection schemes have been described that can (*a*) decrease the amount of time for sets of tilt series to be acquired and further boost the throughput of data collection, (*b*) improve the CTF estimation and correction, and/or (*c*) collect data in a way that allows better utilization of high-resolution information (Figure 2).

#### Single versus dual tilt and continuous acquisition.

Tomography data typically suffer from a missing wedge, since samples cannot be tilted to obtain tilt projections in the full range (–90 to 90°) (124). Multi-tilt axis tomography can reduce this missing information from a wedge to a pyramid or cone (112). The Titan Krios (Thermo Fisher Scientific) was designed initially to tilt along two orthogonal tilt axes. However, it was determined that, due to radiation damage, dual-axis tilt series collection did not offer an advantage over single-axis tilt series collection (28, 41). Thus, new-generation microscopes have a single-tilt holder with improved mechanical stability. Using a single-tilt holder, researchers demonstrated that one could dramatically reduce data collection time to minutes by setting the camera to acquire one continuous movie during the tilt scheme (28, 41), with the electron beam blanked when the stage moves between tilt angles and for a preset amount of time afterward to allow the stage to settle; this is referred to as the fast-incremental method (28, 41).

#### Beam shifts reduce acquisition time.

The rate-limiting step for TEM data collection is waiting for minimal stage drift following stage movements due to stage motor vibrations (27). In SPA, it is routine to use beam shifts rather than stage shifts, obviating the need for settling times and thus increasing the number of attainable targets, as a central focus and tracking area is employed and the beam is then tilted to desired target sites for data collection (27). Data collection is >720 micrographs per hour for a K3 using image shift, a roughly 10-fold increase compared to stage shift (111). Post-collection, the beam tilt is estimated and corrected during CTF estimation (167).

The challenge to implementing this method for tomography is that the distance between a central focus and tracking area and the target point changes during the tilt series acquisition. Beam-image shift electron cryo-tomography (BISECT) was able to adapt this beam-image shift for in vitro tomography by determining relevant geometric constraints (16). BISECT reduced tilt series acquisition time to mere minutes, without compromising data quality, when comparing STA results to collection by the DS method (16). In multishot tomography, researchers implemented beam-tilt image shift by acquiring a multishot tilt series along the tilt axis (77). Unfortunately, cellular features of interest within a lamella are not likely to be arranged neatly along the tilt axis. To overcome this problem, a third group developed the parallel cryo-electron tomography (Pace-Tomo) acquisition scheme, which utilizes a geometric model of the lamella that accounts for the lamella pretilt and sample geometry, and also developed an empirically determined defocus ramp that reduces errors in the tilt axis offset (42). With both Pace-Tomo and multishot tomography, researchers achieved 8 Å reconstructions of an in situ ribosome (16, 42).

#### The best of both worlds? Hybrid subtomogram analysis and single-particle analysis.

In tilt series collection, a total electron dose of 80–200 e^−^/A^2^ is divided along the entire tilt range. This results in a low SNR for each tilt image, since 1–3 e^−^/A^2^ is spent on a tilt image, depending on total dose, tilt increment, and tilt range. A few hybrid STA-SPA (hSTA) workflows have been described wherein a higher-electron-dose image is acquired at the 0° tilt angle, followed by a DS tilt series for the remaining projections (121). The initial high-dose image at 0° has a higher SNR, resulting in a better defocus estimation and CTF correction. Using hSTA, Sanchez et al. (121) improved the resolution of the Tobacco Mosaic Virus (TMV) from 7.2 Å to 4.4 Å compared to a DS tilt series. Similarly, TYGRESS implemented a hybrid workflow: At 0°, the specimen is exposed to an initial high dose (30 e^−^/A^2^), followed by a regular low dose; a DS tilt series is collected afterward (131). In subsequent STA, the high-dose image is used for CTF estimation.

#### Capturing the full picture (montage data collection).

The choice of magnification, and thus field of view, is a compromise between the area that needs to be included in the tilt series and the pixel size, which determines the limit to resolution (151). Another consideration is that higher magnification also results in higher total doses: The dose in an individual image increases as 1/*r*^2^, where *r* is the pixel size. For research inquiries that would benefit from a large field of view without decreasing magnification or resolution, it would be advantageous to obtain one exhaustive tomogram of the entire lamella. This idea was initially developed for tomography of data collected from resin-embedded samples (113). By collecting cryo-ET data composed of overlapping fields of view, two studies tiled multiple images to create one massive montaged tomogram with a large field of view (110, 161).

#### 2D template matching.

**Figure 3:**
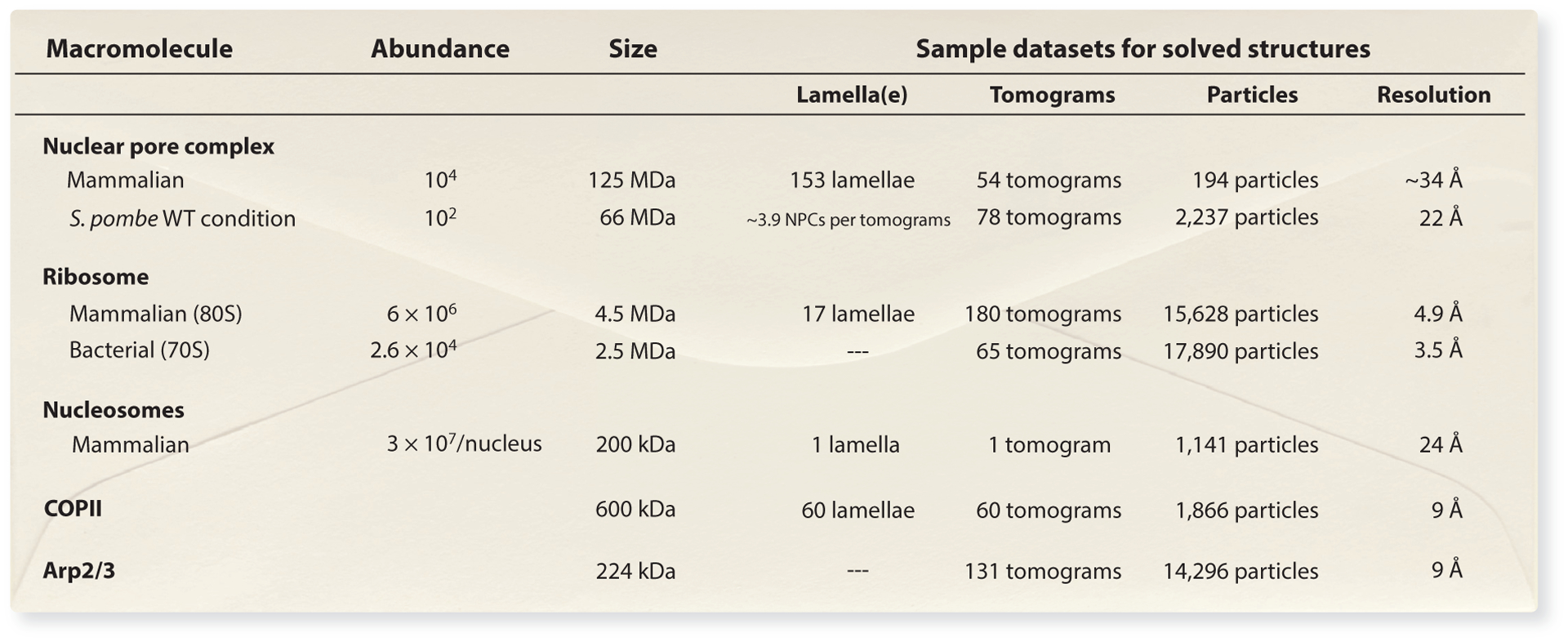
Cryo-ET by the numbers. The size and cellular abundance of a given macromolecule (or its local concentration) are relevant when estimating how many lamellae, tomograms, and particles you will need to perform STA. Not surprisingly, the more abundant protein complexes (e.g., ribosomes, nucleosomes) will have a greater ratio of particles to tomograms compared to less abundant complexes (e.g., the NPC). For the number of NPCs in yeast, see Reference 155; particle numbers and estimated resolution from EMDB 11373 (166). For the number of NPCs in mammals, see Reference 109; particle numbers and estimated resolution from EMDB 12814 (126). For the number of nucleosomes in the mammalian nucleus, see Reference 45; particle numbers and estimated resolution from EMDB 6949 (23). For the number of bacterial ribosomes, see References 59 and 142; BioID 106861; particle numbers and estimated resolution from EMDB 11650 (137). For the number of mammalian ribosomes, see Reference 160; BioID 106861; particle numbers and estimated resolution from Reference 78 and EMDB XXXX (yet to be released). COPII particle numbers and estimated resolution from EMDB 3720 (38). Arp2/3 particle numbers and estimated resolution from EMDB 11869 (48). Abbreviations: ET, electron tomography; NPC, nuclear pore complex; *S. pombe*, *Saccharomyces pombe*; STA, subtomogram analysis; WT, wild type.

For in situ STA, the ability to obtain high-resolution structures, or to unveil multiple conformational and compositional states, can be limited by the number of particles that can be extracted from the data. This limitation can be due to abundance of the macromolecule of interest in cells, the number of in situ conformational or compositional states (e.g., transient interactions), or other factors. Even more challenging is how to find these particles in the crowded environment of the cell, where most proteins have similar shapes and molecular weights. How can we identify our particles of interest and generate high-resolution reconstructions? One powerful idea is to avoid tilt series acquisition and instead acquire a low-defocus and low-dose (approximately 30e^−^/A^2^) 2D projection (120). Using the high-resolution information, users search for particles by template matching in two dimensions, determining the position in X-Y and orientation within the sample as defocus estimation is used to determine the localization along the optical axis (Z). Once these parameters are determined, the particles in two dimensions can be used for averaging to obtain high-resolution 3D reconstructions (89). In a follow-up study, Lucas et al. (90) used this method to locate ribosomal subunits in the nucleus of a yeast cell and found that homolog templates were enough to find relevant particles, with which they could identify and resolve 60S maturation intermediates in situ. Expanding this idea to the entire lamella, in the defocus correction large area cryo-EM (DeCo-LACE) method, a large field of view was collected at low defocus values, enabling the identification of ribosomes at low defocus within the cell by 2D template matching (44). This powerful technique promises to be the key to locating proteins in crowded environments and using high-resolution information to determine their structures, provided that there are templates to search for and that enough particles can be found within the data (see Figure 3).

#### Phase plate.

In TEM, contrast is generated by defocusing the objective lens, and spatial information is recovered by estimating and correcting the CTF. The Volta phase plate (VPP) generates phase contrast by introducing a phase shift between the scattered and unscattered electron waves (35). Since the VPP is a physical material that is exposed to the EM beam, the phase shift evolves over time and needs to be monitored (35). Examples of VPP use for enhancing contrast for tomographic studies include initial work visualizing the nuclear periphery by cryo-FIB-ET (92) and structural analysis on in situ chromatin (23, 50). The VPP does not reliably outcompete standard methods for high-resolution data determination (139). However, it has excited the field due to the possibilities of phase contrast imaging in TEM. It was recently demonstrated that the laser phase plate, a high-intensity continuous-wave laser beam, is capable of generating phase contrast imaging in TEM (128). Because the phase shift is generated by a laser and not a material, continuous exposure to the TEM beam would not affect data acquisition over time.

#### Scanning transmission electron microscopy.

What if we were able to acquire more than just projection images, i.e., more than just a series of gray values from our biological samples? Pioneering work using cryo-scanning TEM (STEM) has demonstrated that it is feasible to obtain spectroscopic information from biological samples (156). STEM uses a weakly convergent beam at high energy and generates an electron diffraction pattern that is then detected by bright field, annular dark field, or high-angle annular dark field detectors (43). As a proof of principle, STEM with integrated differential phase contrast mode resolved TMV to 3.5 Å but imaged with only 30 e^−^/Å^2^ (85). Such low doses would be ideal for in situ tomography.

### Data Reconstruction and Analysis

Interpreting a tomogram is challenging because of the low SNR; recall that biomolecules are weak-phase objects, composed of elements of similar atomic masses, and are radiation sensitive. Additionally, biomolecules exist in a very crowded medium, and many of them have very similar overall shapes. Unequivocally identifying individual proteins or biomolecular complexes is challenging, and the type of analysis that must be performed depends on the focus of the study. Cryo-ET can be used as an imaging method or driven by structure determination, whereas STA is used to determine the architecture of biomolecular complexes in situ. Computational algorithms improve the interpretability of a reconstructed 3D tomogram, facilitating data annotation (segmentation) and particle identification for STA.

#### Reconstruction methods.

After tilt series acquisition, tilt images are aligned and then reconstructed to generate a 3D volume or tomogram; typical methods include weighted back project (WBP) (118), simultaneous iterative reconstruction technique (SIRT), or simultaneous algebraic reconstruction technique (SART) in Image Modeler (IMOD) (100). In WBP, the 2D projection images are back-projected with a weighting function that preserves the high-frequency information that is necessary for further structure determination from the resulting 3D tomogram (57).

The quality of the reconstruction will depend on the accuracy of the tilt series alignment and the accurate correction of local motion. In situ cryo-FIB-ET cannot easily utilize fiducials to solve the tilt series alignment, as with in vitro tomography; thus, recent solutions for image misalignment work computationally. Warp and AreTomo (Alignment and Reconstruction for Electron Tomography) (165) solve for and correct for in-plane rotations, translations, and local motion. While Warp utilizes individual particles averaged from a tomogram to improve the tilt series geometry, AreTomo solves these parameters and applies those solutions to the whole tomogram.

Denoising filters, deconvolution, machine learning (ML), and deep learning (DL) algorithms improve the interpretability of noisy cryo-EM data by acting on either the input 2D projections, during the reconstruction process, or the 3D reconstruction. Denoising filters enhance the SNR and visual interpretability of whole tomograms; these filters include the non-local means filter, median filter, Sigma filter, and Gaussian filters (55). SIRT and SART filters enhance low-frequency information that is suitable for visual interpretation (147). Additional post-reconstruction filters include the non-linear anisotropic diffusion filter, which smooths and enhances edges (56).

ML and DL algorithms, which were developed for restoration of any type of image, have been adopted by denoising programs specifically for EM data. ML strategies such as Noise2Noise, Noise2Void, and Noise2Self are particularly useful when the ground truth is unknown—as it is for cryo-EM data (11, 18, 86, 137). Programs that denoise TEM projection images or tomographic volumes through DL include Warp, CryoCARE, Topaz-Denoise, and IsoNet (11, 19, 87).

#### Deconvolution for missing wedge mitigation.

Due to the inherent geometry of a lamella slab and data acquisition, there is a missing wedge of information in the *Z*-direction, and data collection is limited to +/–60° due to the sample holder tilt range (124). The inability to correct for this missing information, or to account for its absence, can cause artifacts in downstream analysis. Deconvolution is regularly applied to fluorescence microscopy images (122) and, more recently, STEM cryotomography (150). Croxford et al. (31) found that applying an entropy-regularized deconvolution algorithm to cryo-ET data improved the reconstruction by modeling the point spread function (PSF) of the TEM beam in three dimensions, then deconvolving the back-projected 3D image with the 3D PSF to fill the missing wedge, resulting in a less isotropic reconstruction. Using experimental and synthetic data, they demonstrated that their deconvolution method fills in information in the missing wedge lost during either tilt series acquisition or WBP.

Missing wedge mitigation is also performed via isotropic reconstruction of electron tomograms with DL by IsoNet (88). IsoNet adds Gaussian noise to a training set and then iteratively improves the SNR by attempting to eliminate the added noise through DL (88). The information learned during training is then used to fill the missing wedge (88). Standards for denoising tomograms by DL would be helpful as this field progresses, as would guidelines for appropriate user implementation during data analysis. Conservative uses of a tomogram denoised through ML include visual interpretation and particle picking, always referring to the original data for further STA.

#### Data annotation.

After improving the contrast and SNR of a tomogram, annotation enhances the interpretability by defining the relevant features. The process of annotating features in a tomogram is referred to as segmentation. Membranes and filaments are continuous 2D and 1D objects that are readily identifiable in tomograms. Manual membrane segmentation of cellular organelles was initially traced by hand, a time-consuming process. Algorithms that automate at least part of this process include TomosegmemTV (95), Membranorama (153), and the Surface Morphometrics toolkit (5). A growing number of programs identify and annotate cellular features through artificial intelligence: DeepFinder identifies macromolecules (104), and MemBrain identifies membranes (83), as well as programs for filament identification (37).Template-free detection and classification of membrane-bound complexes in a tomogram is another useful tool (96). Complete annotation of a tomogram (membranes, filaments, and macromolecules) can be performed in EMAN2 (26). Software to visualize these annotations and utilize embedded analysis tools include Amira and DragonFly (114, 132).

### Subtomogram Analysis

There are several situations in which in situ tomography is an ideally suited technique, including when the goal is to determine the structure of macromolecular assemblies as they exist in their native environment; when the proteins are deeply embedded in their environments and cannot be faithfully reconstituted outside of cells (9, 108, 130, 158); when the assemblies are transient, or multiple compositional or conformational states exist (1, 2); and when contextual information will never be faithfully recapitulated in vitro (e.g., chromatin topology). The molecular architecture of in situ complexes can be resolved by STA and combined with integrative modeling, facilitating the proposal of new structures and models. STA typically entails multiparticle refinement and its predecessor, subtomogram averaging.

#### Subtomogram averaging.

Following tomogram generation and the visual enhancement of choice, if your molecule of interest is present, then you would explore STA methods. We refer readers to more detailed reviews that describe current STA methodology in theory and in practice (116, 117, 147, 164). First, frames are aligned to account for beam-induced motion (BIM). Then, the CTFs of the tilt images are estimated and corrected; however, because tilted images have a defocus gradient, CTF estimation correction must occur in three dimensions in its local position to accurately determine the CTF for a given particle (140). The tilt series are aligned, and a tomogram is reconstructed (99). The next step is particle picking or template matching for molecules of interest, after which subtomograms around that particle are extracted, and those subtomograms are then aligned to a reference (17). Software packages for subtomogram averaging include Dynamo, EMAN2, emClarity, RELION, and StopGap (10, 14, 24, 148). Structures determined using STA on in situ data are being published at an ever increasing pace. Often, the structures obtained test or generate existing hypotheses that can only be addressed in their natural environment. Examples include the nuclear pore complex (1, 9, 105, 166), bacterial and eukaryotic ribosomes (42, 47, 84, 107, 137), microtubules (54, 64, 135), proteasomes (2, 4), LRRK2 bound to microtubules (149), the Arp2/3 complex (48), the COPI coat (22), nucleosomes (23), a bacterial chemosensory array (20), and a bacterial gap junction (152).

#### Multiparticle refinement.

In situ structure determination improved significantly with the implementation of a framework in which relationships between particles within the same tomogram are solved as part of a larger system. Due to BIM and radiation damage, particles appear to move in different trajectories through a tilt series. By determining the trajectories of one set of particles, one can facilitate the alignment and translation of neighboring particles within that system (6, 137). Implementation of multiparticle refinement yielded a 3.4 Å reconstruction of an in situ stalled ribosome (137). Initially referred to and described as constrained single-particle tomography, multiparticle refinement was fully realized when it was implemented through emClarity and Warp/M (6, 69, 137). By solving the particle trajectories of one species, one can improve the trajectories of another; for example, researchers performed STA on bacterial ribosomes and then used the optimized tilt series to improve the trajectories of the target, a jumbo phage nuclear shell, chimallin, thereby improving the final reconstruction (84).

A major source of difficulty in implementing STA is migrating data between software programs. It is nontrivial to properly convert particle coordinates, metadata, etc. (21). It is not uncommon to jump among Dynamo, Warp/M, and RELION; for instance, the in-cell structure of Arp2/3 relied on all of these software packages (48). A collection of scripts that makes the move among Dynamo, Warp/M, and RELION seamless was recently published, a win for all Team Tomo members (21). Additional scripts for converting RELION-v4 starfiles are available via the RELION-4 Github.

#### Making the most out of angular information.

A very powerful algorithm, NEMO-TOC (NEighboring MOlecule TOpology Clustering), utilizes the coordinates and the Euler angles determined from STA to facilitate the clustering and interpretation of neighboring macromolecules such as polysomes along the same messenger RNA chain (74). There is tremendous potential to apply fully utilized refined particle coordinates to map structures and orientations in their native environment. For instance, future applications using the coordinates and refined Euler angles of in situ nucleosomes would advance our understanding of chromatin chain topology directly inside the cell.

## QUANTITATIVE CELL BIOLOGY AND BIOPHYSICAL MODELING

A cellular tomogram, not unlike a painting by a Dutch master, is rich in both detail and contextual information, with diverse equally mesmerizing vignettes (T. Laughlin, personal communication). Where should one begin the analysis? One could solve the ratio of active to inactive proteasomes within a neuron (2) or measure exactly the differences in dimensions and curvature between two organelles (5, 91). Additionally, since bacteria are comparable in size to the light diffraction limit (76), their internal structure and modeling are challenging for traditional light microscopy methods, and thus many unexpected discoveries and molecular mechanisms have been resolved using cryo-FIB-ET (25).

This section highlights a few examples in which in situ tomography data have been used to better model the characters in a cell and how they interact in a quantitative and biophysical manner. This list is by no means exhaustive, but it serves as an illustration of the power of the technology as a quantitative imaging tool. The earliest work performing in situ cryo-ET studies was on the thin cellular regions, which are easily vitrified and thin enough (<300 nm) that the TEM beam could penetrate (9). Later,visualization of actin comet tails in cells infected by the bacterium *Listeria monocytogenes* facilitated biophysical models for actin branching based on X, Y, and Z coordinates determined in their native state (73).Recent work that beautifully captured actin-mediated endocytosis directly provided the basis for biophysical modeling of this fundamental event (130).

Moving further into the cell, cryo-FIB-ET studies have illuminated junctions between organelles where membrane contact sites communicate and exchange contents. Membrane contact sites are sites of lipid transfer, approximately 20 nm structures, and are difficult to resolve by other techniques. The endoplasmic reticulum (ER) contacts many other organelles, as it is the lipid factory responsible for synthesizing structural phospholipids, sterols, and storage lipids (115). What is the molecular basis for lipid exchange? Direct visualization of the molecules between the plasma membrane and the cortical ER (cER), followed by correlative light microscopy EM (CLEM), made it clear that extended synaptotagmins (tricalbins in yeast) form 16–24 nm bridges between these two organelles at regions of high cER curvature and that these bridges are Ca^2+^ dependent (30, 71).

Through STA and integrative modeling, Wozny et al. (158) recently proposed a model for lipid transport for the ER–mitochondrial encounter structure (ERMES) in which three ERMES synaptotagmin-like mitochondrial lipid-binding protein (SMP) domains stack to form a continuous structure or supramolecular organization that facilitates lipid transport between the ER and mitochondria (158).

Cryo-FIB-ET preserves membrane features extremely well and allows researchers to better visualize membrane remodeling events that occur during stress, starvation, and apoptotic conditions. Stress and starvation elicit the de novo formation of an entirely new organelle, the autophagosome. How the autophagosome is nucleated, which membranes supply lipids to the growing autophagosome, and how subsequent fusion with the lysosome or vacuole occurs are open questions. Visualizing and quantifying the entire process in nitrogen-deprived yeast via cryo-FIB-ET led to a few striking conclusions. It was revealed that general macroautophagy does not require cargo targeting. Through nearest-neighbor analysis, it was determined that the phagophore comes in very close contact with many membranes—lipid droplets, the ER, the nucleus, mitochondria, and the vacuole—potentially explaining conflicting reports about which organelles supply the lipids (15). Finally, it was found that the curvature of the phagophore rim changes shape during phagophore growth, which is perhaps related to how the phagophore rim expands (15).

### Observations that Yield More Questions

Cryo-FIB-ET has discovered new biology (25) and likely will continue to do so, as it is currently the highest-resolution imaging method for cell biology capable of capturing intact molecular networks, and because the imaging source is agnostic to the sample, all molecules are observed. In this section, we highlight a few unexpected observations made by cryo-FIB-ET that have raised additional questions.

While investigating the *Plasmodium* life cycle, researchers discovered microtubule protofilament diversity: 13–18 protofilament microtubules, as well as doublets, triplets, and quadruplets in the gametocyte (52). They found a pseudohelical protein within the microtubule lumen of the sporozite, the *Plasmodium* motile spore-like stage; its identity remains unknown (52). De novo structure determination or AlphaFold mining of the *Plasmodium* genome with structural constraints could perhaps yield its identity, thus realizing visual proteomics.

Models of vesicular transport through the Golgi (from *cis-*Golgi, to Golgi stacks, to *trans*-Golgi) have consolidated around the vesicle maturation model. A rather striking STA result determined that there is an array of proteins in the narrow *trans*-Golgi lumen of *Chlamydomonas* (46). These structures show high periodicity, with interactions between *cis-* and *trans*-facing arrays arranged in a zipper-like fashion, and potentially unidentified glycosyltransferases (46). As these proteins occupy the central portion of the Golgi, it suggests that modified cargo is forced into the Golgi periphery by locally narrowing the *trans*-Golgi lumen (46).

Cell biology studies on how the mitochondria undergoes fission implicated the ER in this process, as it appeared from live-cell confocal fluorescent microscopy that the ER encircled the mitochondria (60). However, in situ cryo-FIB-ET showed that mitochondrial fission directly involved actin and septin right before fission (91). Whether the ER could become involved, after actin and septin, and encircle a mitochondria is currently an open question.

### How to Find Your Favorite Macromolecule

Unequivocally identifying a molecule of interest within in situ cryo-ET data remains one of the largest challenges in using this method. When peering at your tomogram, your eyes are drawn to recognizable features: large macromolecular assemblies, lipid bilayers, filaments. Thus, we have a problem—smaller, less recognizable macromolecules are hiding in plain sight, as it is hard to unequivocally identify a molecule only by a tomogram. Tools to identify your molecule of interest include cryo-fluorescence microscopy (cryo-FM), metal-based tags, and molecular tags. This section covers these technologies and what caveats and challenges exist for each.

## CLEM

There are two steps in which fluorescence microscopy is extremely useful in targeting and identifying molecules of interest. CLEM is a well-established tool for correlating fluorescence images and EM data. Fluorescence images taken under cryogenic conditions (cryo-FM) can be used to identify which cells among the ones in the grid contain the feature of interest to generate a lamella, for instance, when thereis heterogenous protein expression, as with a transient transfection; stochastic expression due to lentiviral integration; or a rare cellular event. The resolution of a cryo-FM image is roughly 250 nm in X-Y and 500 nm in Z (36). As the desired lamella thickness is 100–300 nm, it is possible to mill through your target if you are relying on fluorescence images limited to 500 nm. The second CLEM step overlays the fluorescence image on a low-magnification TEM image of the lamella to identify molecules or features of interest. Unfortunately, correlating protein localizing between fluorescence microscopy and the TEM image is challenging, as it requires correlation between features in both, and is thus best applied to membrane-bound targets, microtubule-bound targets, or discrete assemblies rather than dispersed cytosolic targets. Nevertheless, many studies have been made possible only through CLEM. The recent development of CLEM systems installed inside the dual beam FIB-SEM microscope (63) promises a higher yield and throughput of lamellae containing the features of interest, and commercial devices using these systems are now available, including Meteor (Delmic) and iFLM (Thermo Fisher Scientific).

What about superresolution microscopy? Ideally, we would want to locate proteins of interest within a tomogram with single-molecule precision. The challenges with adopting superresolution microscopy methods for cryogenic conditions include sample devitrification from the laser source, the poor numerical aperture of an air objective lens (36), the difficulty of photoactivating a fluorophore at cryogenic conditions, and correlating the TEM and fluorescence data to high precision (125). A proof-of-principle superresolution experiment using single-molecule active control microscopy (SMACM) utilized a photoactivable protein (PAmKate) to perform correlative cryogenic SMACM and cryo-ET (CIASM, pronounced like chasm) to identify the localization of caulobacter proteins (34). One of the main challenges in this work involves heating (and devitrifying) the sample with the laser, which limits the number of fluorophores that can be detected. Using different substrates, Dahlberg et al. (33) managed to increase the density of localizations and improve correlative imaging. Use of this imaging modality in lamellae remains to be implemented, as thermal conductivity in frozen cellular samples is low. Another exciting development in the attempt to fully leverage superresolution capabilities was achieved through a custom-built liquid helium–cooled microscope, which images samples under vacuum conditions. These high-pressure frozen specimens were imaged by cryo-superresolution microscopy, thereby facilitating the study of ultrastructure relationships in intranuclear vesicles, the ER, and chromatin transcriptional domains between two imaging states: superresolution fluorescence and electron microscopy (70).

### Other Molecular Tags

Besides tagging proteins of interest with fluorescent proteins, use of other types of tags that can be visualized directly in the TEM would dramatically expand the amount of the proteome that is accessible to cryo-ET. One possibility is to use signpost origami tags (SPOTs), which recognize and bind green fluorescent protein (GFP) through an aptamer composed of DNA origami (68). The effective distance from the target to the SPOT is roughly 70 nm, which may or may not be achievable and may disrupt the molecular networks, depending on the query.

Using nanoparticles composed of atoms with high atomic numbers to find your molecule of interest would be ideal, as biological material is mostly made of the lighter elements—carbon, nitrogen, oxygen, phosphorus, trace metals, etc. Heavy elements such as gold or iron would appear as a darker contrasting object, as the atom’s nucleus is more dense by comparison to the biological sample and would scatter more electrons from the imaging source. An example of this is the FerriTag system, in which the molecule of interest is expressed as a fusion construct to GFP-FKBP, and dimerization occurs when rapamycin is supplied and FKBP binds to a coexpressed fusion of an FRB-mCherry-ferritin cage (29).The large ferritin cage is roughly 40 nm away from the molecule of interest (29).Another ferritin-based labeling strategy expressed a ferritin cage within *Escherichia coli* and was used to target specific proteins of interest (146). Additionally, a 2.2 nm nanogold particle was conjugated to designed-ankyrin repeat proteins (DARPins), which were engineered to recognize extracellular integrins on a mammalian cell and visualized by cryo-ET (32).

## CRYO-ELECTRON TOMOGRAPHY BY THE NUMBERS

Above, we describe technical hurdles in cryo-ET and relevant solutions that allow us to capture macromolecules in near-native conditions; however, a greater challenge is selecting or designing the right model system. The scientific questions driving a project will determine how the data are acquired, what kind of analysis is performed, and what amount of data is required. If structure determination is the goal, then what is the resolution required? How abundant is the complex or event of interest in the cell, and thus, what is the likelihood of capturing it in a tomogram? If it is present, can it be unequivocally identified? What is the potential conformational and compositional variability of the protein or complex? The bottom-line question is how many instances of an event must be captured to better understand its structure–function relationship in situ? From this question follows another: Is in situ absolutely required?

Let us consider the fact that a typical mammalian cell is roughly 2,000 μm^3^ (103) (BioNumber Identification 108244). A typical lamella is a 3D slab that is ideally approximately 100–200 nm in thickness and covers an area of approximately 10–20 μm per side (approximately 100–400 μm^2^); thus, the lamella volume is approximately 10–80 μm^3^, covering 0.5–4% of the cell. With the magnifications typically used for cryo-ET, which result in an imaging area of 0.4 to 2 μm per side, a tomogram covers significantly less than a percentage of the total cell volume. Knowing the total copy number (or the local concentration) of your protein of interest is necessary to obtain an estimation of how many lamellae, tomograms, and particles will be necessary to observe your chosen macromolecule and perform relevant analysis. To answer the question of how many lamellae and tomograms are needed to answer your question or solve a structure, estimate how many copies one could reasonably achieve per tomogram, how heterogeneous these instances are expected to be, and what resolution is necessary to answer the question. The size and cellular abundance of a given macromolecule (or its local concentration) are relevant when estimating how many lamellae, tomograms, and particles you will need to perform STA. Not surprisingly, the more abundant protein complexes (ribosomes, nucleosomes) will have a greater ratio of particles per tomogram compared to less abundant complexes (the nuclear pore complex). However, since the nuclear pore complex and the ribosome are megadalton-sized complexes, they can be easily identified in a crowded tomogram, and their large size also facilitates particle alignment during STA. Such back-of-the-envelope calculations are useful when estimating how much data (roughly) are required to answer a given question (see Figure 3).

## THE STRUCTURAL REVOLUTION

Before the resolution revolution in SPA (81), integrative modeling was used to bring together data from various sources (X-ray, cryo-EM, mass spectroscopy, cross-linking, proteomics, and others) to model the architecture of macromolecular complexes. This tradition has continued as cryo-ET has begun to produce maps of challenging complexes in situ (1, 149). More recently, the advent of AlphaFold has potentialized this methodology: Models from AlphaFold can be used to interpret the cryo-ET data, e.g., using template matching, and to assist in building molecular models from in situ maps obtained through STA (53, 106).

## OUTLOOK

Cryo-ET has rapidly established itself as a powerful technology to bring structure to cell biology. Although lacking in temporal resolution, this technique is currently the highest-spatial-resolution imaging tool available for cell biology, and it can be used to make new discoveries or to generate new hypotheses that can be further tested with other methods such as live cell imaging and proteomics. It can also produce novel structures of macromolecular complexes embedded in their environment or perform censuses on the number and composition or conformation of molecular complexes in cells, as well as other quantitative analysis. Future advances and automation will democratize this technology and allow it to become a widely available and powerful tool for the cell biologist.
